# Cold Water Enemata in Cholera Infantum

**Published:** 1888-11

**Authors:** 


**Cold Water Enemata in Cholera Infantum.**—Dr. Bailey writes the *Southern Clinic*, that it is useful to give ice water freely, but it is far better to give cold water enemata, and thoroughly cleanse out the pernicious and offending matter from the intestines, and this done, the trouble ceaes at once. It has been my experience for many years in bowel troubles to resort to this most potent remedy, and I have never found it to fail. As a rule medicine is not required, but, if it is given, it should be such as to allay irritation and not of an astringent nature.

